# Unexpected Different Binding of Mistletoe Lectins from Plant Extracts to Immobilized Lactose and N-acetylgalactosamine

**Published:** 2007-09-17

**Authors:** Tibor Hajtò, Fodor Krisztina, Aponyi Ildikò, Pallai Zsolt, Balogh Pèter, Németh Pèter, Perjési Pàl

**Affiliations:** 1 Department of Medical Chemistry, University Medical School of Pécs,; 2 Department of Immunology and Biotechnology, Faculty of Medicine, University of Pècs, Hungary.; 3 Dachen Kft, Budapest

**Keywords:** FPLC, mistletoe extract, mistletoe lectins, Viscum album L

## Abstract

Mistletoe Extracts (ME) are of growing interest to pharmacological research because of their apoptosis-inducing/cytostatic and immunomodulatory effects. The standardization of the three different groups of Mistletoe Isolectins (ML-I, II and III) is often rendered more difficult since the primary structures are nearly identical. Their classification is based on their Galactose- and N-acetyl-D-galactosamine (GalNAc)-specificity which was measured by various inhibitory assays. The aim of the present study was to improve the characterization of the direct binding activity of the isolectins from ME to immobilized lactose, GalNAc and to the oligosaccharide asialofetuin. After careful ultrafiltration of fresh ME, affinity chromatography was carried out using lactose- agarose, GalNAc—agarose and asialofetuin—affigel 15 columns. MLs were further purified by Sephadex G-100 or by cation exchange chromatography which was adapted to a Fast Protein Liquid Chromatography (FPLC) system. Proteins from both fresh plants and commercial ME were able to bind immobilized lactose to a considerable extent. The majority of this lectin has a B-chain with a Molecular Weight (MW) of 34kD and an A-chain with a MW of 29 kD (ML-I). Only a minor part of the lactose-binding proteins has a lower MW, namely 32kD and 27kD (MLII). However, neither MLs which were eluted from lactose columns, nor the proteins from fresh plant or ME showed a direct binding to the immobilized GalNAc. In spite of this deficiency, GalNAc was able to induce a considerable (25% and 32%) inhibitory effect on their binding to immobilized asialofetuin indicating a discrepancy between the lectin binding and inhibiting effects of GalNAC. Consequently, for an improved standardization of ME more specific sugar molecules are necessary.

## Introduction

In the standardization of commercial mistletoe extracts, the correct determination of mistletoe lectins plays an important role. However, plant mistletoe lectins exhibit a heterogeneity which most likely results from the posttranslational processing of Mistletoe Lectins (ML)-I to the isoforms ML-II and ML-III [Eck, 1999] since only a small difference was found in their primary structure. The antigenic analysis of B- subunit in ML-I and ML-III showed one epitope ^25^RDDDFRDGNQ^34^ in ML-I that is absent in B chain of ML-III and this difference can be related to a little gene polymorphism [Pevzner, 2004]. Mistletoe extract preparations vary with regard to the content of ML-isoforms, which also may depend on the method of isolation or on various degradation effects. Growing evidence suggest that immunomodulatory efficacy of mistletoe extracts (ME) is dose-dependent with a bell-shaped dose-curve and correlates with the lectin-sugar interactions on the membrane of various immune cells [Hajto, 1989a; Hajto, 1989b]. However, it has been criticized that mainly ML-I was taken into consideration instead of ML-II or ML-III for immunological research and standardization of ME [Jäggy, 1995]. The chemical definition of ML-II and III is based on the lower molecular weights, the small differences in primary structures and on the observation that N-acetyl-D-galactosamine (GalNAc) exhibits a more marked inhibition on ML-II or ML-III-induced hemagglutination or cytostatic activity than the galactose-specific ML-I. However, few data are available concerning binding capacity of ML-II and ML-III to immobilized GalNAc. In the present study, the aim was to minimize degradation effects during the separation. Therefore, mistletoe lectins were carefully isolated from fresh plants and from commercial mistletoe extracts by ultrafiltration and using affinity chromatography. The direct binding capacity of MLs to lactose and to GalNAc was compared in the same system. Surprisingly, no direct binding to immobilized GalNAc was detected. Only immobilized lactose was able to bind MLs from our specially prepared extract. These unexpected findings can be interesting for the further research of MLs which requires new perspectives to find appropriate ligands with considerably higher affinity for MLs, such as CD75 gangliosides as described several years ago [Müthing, 2002]. Since CD75 oligosaccharides can exist on a great number of effector cells of innate immune system [Kawashima, 2003], MLs may be important candidates for an immunotherapy with targeting strategy.

## Material and Methods

### Material

Mistletoe plants freshly picked from apple trees during the winter months when the lectin content of leaves and stems is high [Urech, 2001] were kindly provided by Dr. Urech (Society for Cancer Research, Switzerland). Commercial Mistletoe Extract (M spec 200 mg 4125 SF 0404) was kindly provided by Dr. M. Werner (Society for Cancer Research, Switzerland).

### Ultrafiltration

To avoid possible denaturation by ammonium sulfate precipitation during the isolation of MLs, a Sartocon Mini Crossflow ultrafiltration system was used which allows a gentle preparation using low pressure and temperature as well as a short procedure. After extraction of fresh plants (leaves and two terminal stems) in phosphate buffered saline (PBS) solution at 4 °C overnight. To remove the solid particles the suspension was filtered and centrifuged at 15′ 000 g for 7 min and then the pellet was discarded. Using a Watson Marlow pump, the supernatant was ultrafiltered in a Sartocon Mini Crossflow system (Sartorius GmbH, D-Göttingen) through a cellulose acetate filter (Typ 303 145 49 01E) with 20′ 000 NMGT. Based on the instructions of the manufacturers, the slow speed of the pump (43 rpm) guaranteed a low pressure (<0.2 bar) during the procedure. After circulation through the filter for one hour a protein concentrate was obtained with a MW >20 kD.

### Affinity chromatography

Alfa-lactose agarose was purchased from Sigma Aldrich GmbH (Switzerland) and the column (0.8 × 11 cm) was pre-equilibrated with PBS. The flow rate was 15 ml/hr. The elution was carried out with 0.2 M lactose and the eluted fractions were checked using a Specord M40 spectrophotometer (Zeiss, Jena, Germany) at 280 nm. Each fraction contained 1.5 ml elute allowing a kinetic judgment of the elution process. The eluate was dialyzed against PBS and/or bidistilled water and concentrated to 1 mg/ml protein (Lowry method) using Microcon YM-30 tubes (Millipore, Switzerland). GalNAc—agarose column (SigmaAldrich GmbH; Switzerland) was prepared and used in a similar manner as described for lactose—agarose. The elution was carried out with 0.2M GalNAc and 1.5 ml fractions were examined with a spectrophotometer against 0.2M GalNAc. An ultrafiltrate of ME from fresh plants was also passed through an affinity column of asialofetuin (Sigma Aldrich GmbH, Switzerland) bound to Affigel 15 (Bio-Rad Laboratories AG, CH-Reinach) according to the method described by the manufacturer. This column was first equilibrated with equilibration buffer (0.1M Tris HCl, 0.5M NaCl, pH7), before passing the ME ultrafiltrate through at a flow rate of 4ml/min. The column was washed with the equilibration buffer until there was no detectable absorption at 280 nm in the eluate. Then the adsorbed proteins (MLs) were eluted with 0.1M glycine buffer (pH 3). The pH of the fractions was immediately increased neutralized with 1M Tris-HCl buffer (pH 7). Sephadex G-100 was purchased from Sigma Aldrich GmbH (Switzerland) and the column (0.8 × 60 cm) was pre-equilibrated with TRIS buffer (pH 7.5) containing 0.2M NaCl before washing with PBS. The eluate from the lactose agarose column was added to this Sephadex G-100 column. The colunn was then eluted at a flow rate of 10 ml/hr and collected in volumes of 0.5 ml. The affinity chromatography procedures were carried out at 4 °C.

### Fast protein liquid chromatography (FPLC) separation of eluted MLs using cation exchange chromatography

The eluate from the affigel-asialofetuin column was dialysed against 15 mM citrate buffer (pH 4.2), then concentrated to 2 mg/ml using Microcon YM-30 tubes (Millipore, Switzerland) before applying to a cation exchange chromatography column (Mono S 5/50 GL; Amersham Bioscience, Switzerland). The column was adapted to a FPLC system and equilibrated with citrate buffer (pH 4.2). MLs were eluted with a linear NaCl gradient from 0.2 to 0.5M in citrate buffer.

### Electrophoresis

Polyacrylamide gel electrophoresis was performed according to conventional methods [Laemmli, 1970; Ziska, 1993] using 10% polyacrylamide with sodium dodecyl sulfate (SDS) in 1.5M Tris buffer (pH 8.8). The running buffer was Tris/Glycine (pH 8.3) and SDS. The gels were stained with comassie blue. Mercaptoethanol was used as reducing agent.

### Enzyme linked lectin assay (ELLA)

The binding capacity of isolated mistletoe lectins from plants and extracts to asialofetuin was carried out with an optimized ELLA technique as described previously [Hajto, 1989a]. Briefly, the method is based on binding of lectin to an immobilized oligosaccharide ligand (asialofetuin) and subsequent binding of specific (polyclonal) antibody to the bound lectin. The specific binding of rabbit antibodies was quantitatively assessed using goat anti-rabbit peroxidase and the subsequent generation of a colored product from the substrate phenyl-endiamine hydrochloride. The measurements were carried out in an enzyme-linked immunosorbent assay plate reader at 492 nm. After its isolation from fresh mistletoe plant, the standard galacto-side-binding mistletoe lectin was previously lyophilized. The solubility of this standard material was improved by addition of non-specific sugars and its amount was determined gravimetrically.

## Results

### Binding of mistletoe protein concentrate to immobilized lactose and GalNAc

**Figure 1:**
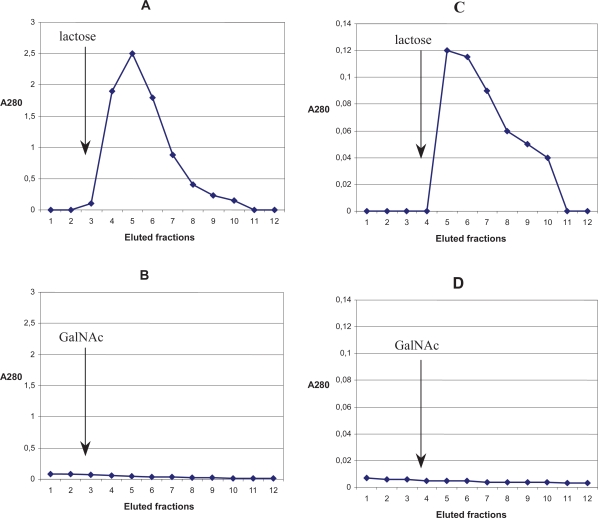
Kinetic representation of lectin content in 1.5 ml eluted fractions measured spectro-photometrically after affinity chromatography of fresh mistletoe plant (leaf and stem) ultrafiltrate on lactose-agarose column (**A**) and on GalNAc-agarose column (**B**) or after the same affinity chromatography of commercial mistletoe extract (Iscador^R^ M-spec) on lactose-agarose column (**C**) and on GalNAc - agarose column (**D**) indicating a lack of binding of mistletoe proteins to immobilized GalNAc.

The binding of fresh plant MLs to immobilized galactose or GalNAc was first examined. The ultrafiltration of fresh plant aqueous ME followed by affinity chromatography allowed a gentle isolation of the protein (ML) concentrate. To investigate the binding of this concentrated protein to immobilized sugars, lactose-agarose and GalNAc-agarose affinity chromatography columns were run in parallel under the same conditions. Figure 1 represents the UV absorbtion of the eluted fractions measured spectrophotometerically. As demonstrated in Figure 1-A, the addition of 0.2M lactose to lactose-agarose column caused a prompt elution of abundant galactoside-binding mistletoe lectin. However, no GalNAc—specific lectin was eluted on the other column (Fig. 1-B).

Among the carefully isolated plant proteins, no lectin which bound directly to immobilized GalNAc could be detected. In addition, lectins eluted from lactose-agarose on GalNAc—agarose column were also tested and no direct binding to this sugar was detected (data not shown).

### Comparison of the binding of mistletoe proteins from commercial extracts to immobilized lactose and GalNAc

As previously shown [Jäggy, 1995], commercial ME has been shown to contain detectable amount of ML-II and ML-III and their agglutination-inducing and cytotoxic effects (ML-III>MLII) with GalNAc were more strongly inhibited than with lactose or galactose [Jäggy, 1995; Franz, 1981; Ziska, 1993; Frantz, 2000]. Therefore, the direct binding of ME proteins to immobilized sugars was also tested using affinity chromatography. As shown in Figure 1-C, after affinity chromatography on the lactose-agarose column, the elution with lactose resulted in a prompt occurrence of galactoside-binding mistletoe lectins. However, as with fresh plant extract on a GalNAc—agarose column, no GalNAc—binding lectin was eluted (Fig. 1-D). The binding of ML-III may require a different spacer for the binding of terminal GalNAc to ML.

### Characterization of galactose-binding mistletoe proteins by electrophoresis and by affinity chromatography on Sephadex G-100

**Figure 2:**
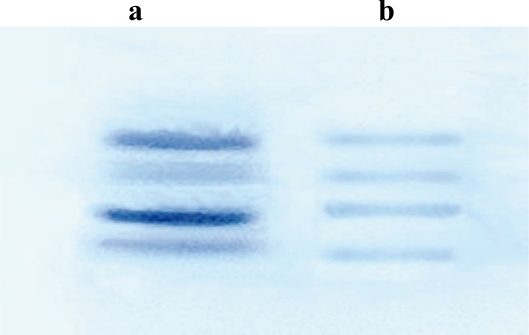
Polyacrylamide gel electrophoresis of lactose-binding lectins isolated from fresh mistletoe plants (**a**) and from commercial mistletoe extract, Iscador M-spec (**b**) after a treatment with reducing agent. The molecular weight of lectin bands from above down wards are the following: 34kD, 32kD (corresponding to B chains of ML-I and ML-II/III, respectively) as well as 29kD and 27kD (corresponding to A chains of ML-I and ML-II/III, respectively).

As shown in Figure 2-A, the majority of galactose-binding mistletoe lectins which were isolated by affinity chromatography consisted of a 34kD B-chain and an A-chain of 29kD. Although MLs were not quantitatively isolated, small amounts of another molecule with 32kD and 27kD chains appear to be present, which may correspond to MLII/III. MLs from mistletoe extract isolated by affinity chromatography on lactose-agarose column show a similar pattern in electrophoresis (Fig. 2-b).

For more exact examination of this heterogeneity, MLs isolated from fresh plants using affinity chromatography on sephadex G-100 were also tested. These investigations are not conclusive, but the first kinetic measurements of 0.5 ml eluted fractions by spectrophotometer also point to only a slight heterogeneity in the molecular weights of these lectins (data are not shown). Further investigations are in progress.

### Inhibitory effect of lactose and GalNAc on the direct binding of MLs isolated from fresh plants and from commercial mistletoe extract to immobilized asialofetuin using ELLA

**Figure 3:**
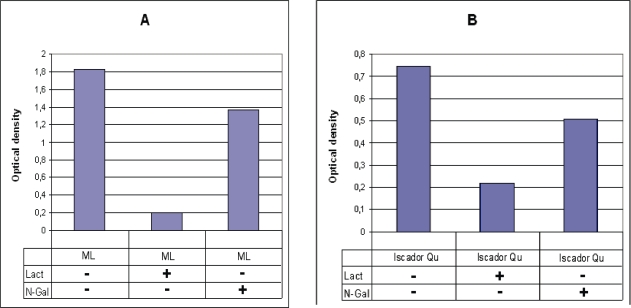
Inhibitory effect of lactose and GalNAc on the binding of MLs to immobilized asialofetuin using ELLA method. Lactose binding lectin from fresh plant (corresponding to 650 ng lyophilized standard lectin) was investigated by ELLA in presence of 0.1M lactose and 0.1M GalNAc (**A**). Similar investigations were carried out with Iscador^R^ Qu-special the lectin content of which corresponded to 335 ng standard (**B**).

Since immobilized GalNAc was not able to bind proteins from fresh mistletoe plants or commercial extract, the inhibitory effect of this sugar on the direct binding to immobilized asialofetuin was also investigated using the ELLA method. As shown in Figure 3-A, MLs isolated from fresh plants by affinity chromatography on a lactose-agarose column in the presence of 0.1M lactose revealed 89% decrease in the binding of MLs to asialofetuin. Surprisingly, GalNAc was also able to induce a 25% inhibition (Fig. 3-A) in spite of the fact that no direct binding of these MLs to GalNAc was detected using affinity chromatography. Similarly different inhibitions by lactose and GalNAc (72% and 32% decreases, respectively) were observed with commercial mistletoe extracts (Fig. 3-B).

### Separation of mistletoe lectins by cation exchange chromatography adapted to FPLC

**Figure 4:**
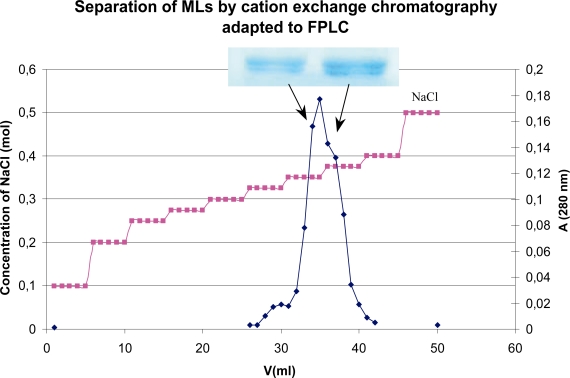
Separation of MLs by cation exchange chromatography adapted to FPLC. Using linear gradients from 0.1M until 0.5M NaCl, the fractions of MLs were eluated from Mono S 5/5O GL column. The small first peak at 0.325M NaCl concentration may represent MLII / III and the second peak between 0.35 and 0.375M NaCl level could correspond to ML-I.

Since MLs are positively charged because of their NH_3_^+^ cations, cation exchange chromatography adapted to FPLC was selected for their further separation. As shown in Figure 5, using linear gradients from 0.1M to 0.5M NaCl, this method also resulted in a small heterogeneity of fresh plant MLs eluted from the Affigel-Asialofetuin column. The rather small first peak at 0.325 NaCl concentration may represent MLII and III followed by a larger second peak between 0.35M and 0.375M NaCl that could correspond to ML-I. However, the further separation of MLs was not successful and the electrophoresis also did not support this hypothesis since the fractions originated from the large second peak of FPLC were also not homogeneous (Fig. 4).

## Discussion

Recent investigations with mistletoe lectins from fresh plants and extracts suggest that new preparation techniques may lead to novel chemical entities which are not identical with the previous results [Franz, 1981; Ziska, 1993; Frantz, 2000]. On the base of earlier procedures with affinity chromatography, three important groups of isolectins were repeatedly identified: D-galactose-specific ML-I, D-galactose and GalNAc-specific ML-II as well as GalNAc—specific ML-III [Franz, 1981; Ziska, 1993; Frantz, 2000]. Further differentiation of these MLs was based on their different MWs and on the differences in the sugar-induced inhibition of their cytotoxic or agglutinin activities. In contrast to these data, several investigators established only two types of MLs (galactose—and GalNAc-specific ML-I and II) in various mistletoe extracts [Samtleben, 2001]. Consequently, in the present experiments, the lack of the binding of proteins from mistletoe plant and extract to immobilized GalNAc is unexpected. For isolation of MLII and III various methods were used. Previously, a protein concentrate from ME was usually obtained using ammonium sulfate precipitation followed by isolation of MLs using acid-treated Sepharose or Affigel-15-asialofetuin column for affinity chromatography. MLII/III were separated by various methods employing immunoglobulin—sepharose and sepharose-N (6-aminohexanoyl)-D-galactosamine [Jäggy, 1995] or sephadex G-100 [Ribereau, 1997] or cation exchange chromatography columns [Frantz, 2000; Eifler, 1993]. In the present experiments, MLs were carefully isolated by ultrafiltration and by affinity chromatography using various agarose—sugar columns keeping the pH, ionic strength and temperature within a constant optimal range during the procedure. However, from the present results it is not possible to make a definitive conclusion and further comparative investigations are necessary to clarify the reasons for the discrepancy between the previous [Ribereau, 1997; Frantz, 2000; Urech, 2006] and present results concerning the binding capacity of ML-III to GalNAc. The binding of ML-III to GalNAc-agarose column may require a different spacer which could better help the binding of terminal GalNAc to ML. Consequently, further comparative investigations are necessary. The most probable hypothesis for this discrepancy may be connected with the discovery [Müthing, 2002] that galactose, lactose and other mono- or disaccharides exhibit only a weak affinity to MLs if they were compared with CD75 receptors which are terminally α2-6-sialylated gangliosides.

The differences in the molecular mass and sugar binding of the two or three species were also thought to be a result of the degree of glycosylation [Zimmermann, 1996]. For example, recombinant ML is not glycosylated and using competitive ELLA, GalNAc was found to have a stronger inhibitory effect on the binding of recombinant ML to immobilized asialofetuin than that of galactose, indicating a potential role for glycosylation in the sugar-induced inhibitory actions [Zinke, 1996]. The complete amino acid sequence of the A- and B-chains of ML-I has been determined [Soler, 1996; Soler, 1998]. The A chain contains 254 amino acid residues and using matrix-assisted laser desorption ionization mass spectrometry (MALDI-MS), the existence of a potential N-glycosylation site was confirmed [Soler, 1996]. The sequence Asn^112^-Gly^113^-Ser^114^ could be identified as a glycosylation pattern. The bound carbohydrate moiety showed a MW of 1173 Da. The B-chain is composed of 264 amino acid residues and three potential N-glycosylation sites were confirmed by MALDI-MS analysis [Soler, 1998]. These are Asn^61^-Gly^62^-Ser^63^, Asn^96^-Gly^97^-Thr^98^ and Asn^136^-Asp^137^-Thr^138^, respectively. The three sugar chains are covalently bound to the protein part of the B-chain and show a MW of 3900 Da. Isolation methods can induce alterations in the glycosylation pattern of MLs [Zimmermann, 1996]. It was also concluded that glycosylation of MLs mainly influences stability, conformational variants, non-specific binding and solubility [Pfüller, 2001; Lutter, 2001].

Many years ago it was established by 2-D gel electrophoresis that there are at least 40 isolectins of MLs [Samtleben, 2001]. For the chemical standardization of the clinically applied ME preparations, the exact determination of isolectin patterns is not possible. The amino acid analysis revealed 17 conservative substitutions along the amino acid sequence of the A-chain. The most noteworthy substitution may be the replacement of Asn^112^ → Thr^112^, which presumably eliminates the glycosylation signal in the amino acid sequence (Asn^112^-Gly^113^-Ser^114^ → Thr^112^-Gly^113^-Ser^114^). These results provide evidence that the A-chain of ML-I has at least two isoforms, one glycosylated and one non-glycosylated [Soler, 1996] which may also correspond to our FPLC result. A small heterogeneity in the peak of ML-I was detected. Analyzed sequence data of B-chain also show 12 conservative substitutions, most of them located in the C-terminal region of the protein. These findings demonstrate that the B-chain, like the A-chain occurs in several isoforms, supporting further evidence for the previously reported existence of different isolectins of ML-I [Soler, 1998].

Because of the heterogeneity of ML isoforms, an exact immunological standardization of commercial ME is not easy. For example, different laboratories using the same methods (cation exchange chromatography adapted to FPLC) found that the ML peaked at dissimilar NaCl concentrations even if the same linear gradients were applied [Frantz, 2000; Eifler, 1993]. Previous studies with ME revealed that the immunomodulatory effects and the sugar-binding activity of an extract have a close relationship [Hajtò, 1990]. Therefore it would be advisable to employ a standard procedure for the exact and reproducible determination of sugar-binding potency of ME using chemically well-defined, stable and reliable lectin standards.
